# Clinical Course of Autoimmune Hepatitis in Hispanic and African American Patients: A Retrospective Study at a South Bronx Hospital

**DOI:** 10.7759/cureus.81082

**Published:** 2025-03-24

**Authors:** Haider Ghazanfar, Franklin Sosa, Raul Reina, Faryal Altaf, Sameer Kandhi, Abhilasha Jyala, Priscilla Lajara, Bhavna Balar

**Affiliations:** 1 Internal Medicine, Bronxcare Health System, New York, USA; 2 Internal Medicine, BronxCare Health System, New York, USA; 3 Gastroenterology and Hepatology, BronxCare Health System, New York, USA; 4 Gastroenterology, BronxCare Health System, New York, USA

**Keywords:** african americans, autoimmune hepatitis, cirrhosis, decompensation, hispanics, south bronx

## Abstract

Background

Autoimmune hepatitis (AIH) is a chronic inflammatory condition that can progress to liver cirrhosis. Genetics, immune system dysfunctions, and environmental factors influence the global prevalence of AIH. AIH exhibits variable clinical outcomes across ethnic groups, with Hispanic patients having a higher prevalence of cirrhosis, whereas African American patients are noted to have higher hospitalization and mortality rates.

Aim

The purpose of our study is to assess the clinical course of autoimmune hepatitis, specifically in Hispanic and African American patients.

Methodology

We performed a retrospective chart review of patients diagnosed with AIH and managed by the Gastroenterology Service from July 2006 to June 2023. The study population comprised individuals who were either Hispanic or African American and aged 18 years or older. Patients who were hospitalized and did not continue with outpatient follow-up were excluded from the analysis.

Results

Out of the 30 patients in our study, 27 (90%) were female and 3 (10%) were male. About 21 (70%) of the patients were Hispanic, while 9 (30%) were African American. The mean age at the time of AIH diagnosis was 45 years. Liver cirrhosis was confirmed with liver biopsy in 21 (70%) of the patients, and by imaging or clinical findings alone in an additional 3 (10%). Concomitant autoimmune diseases were present in 7 (23%) of the patients. Approximately 11 (36%) of the patients required hospitalization due to decompensated liver cirrhosis. About 19 (63%) were initially referred to the gastroenterology service due to abnormal liver function tests and were asymptomatic at the time of the first visit. About 6 (20%) of the patients presented with abdominal pain as their initial symptom. One patient had nausea and vomiting, two presented with jaundice, and one presented with altered mental status. Notably, none of the patients died during the study period.

Conclusion

Our study indicates that AIH is more prevalent among female and Hispanic patients as compared to male and African American patients. A significant proportion of our patients developed cirrhosis. Further studies are necessary to improve outcomes of autoimmune hepatitis in African American and Hispanic populations.

## Introduction

Autoimmune hepatitis (AIH) is a rare liver disease characterized by periportal inflammation, lymphoplasmacytic infiltration, hypergammaglobulinemia, and elevated levels of antinuclear and anti-smooth muscle antibodies. Nonetheless, these features are not definitively diagnostic and can overlap with other liver diseases, including autoimmune biliary disorders. AIH has the potential to progress to chronic liver disease and cirrhosis, affecting individuals of all ages [1].

The prevalence of AIH varies globally, with differences attributed to genetic, environmental, and immunological factors. AIH is believed to result from a genetic predisposition, dysregulated immune responses, and environmental triggers. It can present with a range of clinical manifestations and may remain asymptomatic until advanced stages. By the time of diagnosis, approximately 25% of patients show histological signs of cirrhosis. While AIH can lead to common complications associated with chronic liver disease, the incidence of hepatocellular carcinoma in patients with AIH is generally lower than in those with chronic viral hepatitis [2-4].

Management of AIH primarily involves prolonged immunosuppressive therapy, and treatment discontinuation leads to recurrence in approximately 80% of cases. AIH affects individuals across all races and ethnicities; studies suggest that clinical presentation and outcomes may vary among different groups. Hispanic patients have been identified as having the highest prevalence of cirrhosis at the time of AIH diagnosis, suggesting a rapid progression of the disease. Additionally, hospitalization rates are markedly higher among African American and Latino populations compared with their White counterparts. For African American patients, this increased rate of hospitalization is not only a marker of more frequent inpatient medical care but also has been associated with increased mortality [1,2,5].

The clinical course of autoimmune hepatitis in Hispanic and African American populations is not well understood or widely published in the literature. The purpose of our study is to assess the clinical course of autoimmune hepatitis, specifically in Hispanic and African American patients.

## Materials and methods

Study design

We conducted a retrospective chart review of patients with AIH managed by the Gastroenterology Service at BronxCare Hospital, Bronx, New York, from July 2006 to June 2023.

Inclusion and exclusion criteria

Patients with a confirmed diagnosis of autoimmune hepatitis were included in the study. The diagnostic criteria outlined in the Diagnosis and Management of Autoimmune Hepatitis guidelines published by the American Association for the Study of Liver Diseases (AASLD) were used to establish the diagnosis [6]. Diagnosis required compatible histological findings, elevated aminotransferases, elevated immunoglobulin G, and/or a positive serological marker, along with the exclusion of other diseases that may resemble AIH. The study included Hispanic and African American patients who were 18 years or older. Patients of other ethnicities were excluded, as the purpose of the study was to focus on the progression of autoimmune hepatitis in Hispanic and African American populations. Patients who were seen only as inpatients and did not have outpatient follow-up in our liver clinic were also excluded.

Data collection

All patients who met the inclusion criteria were included in the study. Patient charts were reviewed for demographic data, age at diagnosis, presence of concomitant autoimmune diseases, hospitalization requirements, complications, and medications used in management.

Statistical analysis

Data were analyzed using IBM SPSS Statistics for Windows, Version 21 (Released 2012; IBM Corp., Armonk, New York). The analysis aimed to assess the clinical outcomes and disease progression in our patients.

## Results

A total of 30 patients met our inclusion and exclusion criteria for the study. Of the three male patients, one was Hispanic and two were African American. The demographic characteristics of the study population are presented in Table 1.

**Table 1:** Demographic Characteristics of the Patient

Demographic characteristics	Number of patients	Percentage (%)
Total patients	30	100%
Female	27	90%
Male	3	10%
Hispanic	21	70%
African American	9	30%

The mean age at the time of AIH diagnosis was 45 years. In our study, all African American patients were diagnosed by the age of 52, while 13 (61%) of the Hispanic patients were diagnosed with AIH after the age of 52. The majority of patients developed liver cirrhosis, and a significant number required hospitalization due to decompensated liver disease. Out of a total of 24 patients with liver cirrhosis, 7 had cirrhosis at the time of diagnosis, while 17 developed it as a result of disease progression. About 4 (44%) of the African American patients had liver cirrhosis, compared to 20 (95%) of the Hispanic patients. Approximately 7 (33%) of the Hispanic patients and 4 (44%) of the African American patients required hospitalization for decompensated liver disease. Patients who received steroids were initially prescribed prednisone at a dose of 40 to 60 mg daily, which was gradually tapered to a target dose of 10 mg daily. The clinical outcomes and treatment details of the patients are presented in Table 2.

**Table 2:** Clinical Outcomes and Treatment

Clinical outcomes and treatments	African American (n = 9)	Hispanic (n = 21)
Mean age at diagnosis	38	48
Liver cirrhosis	4 (44%)	20 (95%)
Concomitant autoimmune disease	2 (22%)	5 (23%)
Hospitalization due to decompensated liver disease	4 (44%)	7 (33%)
Treatment with oral corticosteroids	5 (55%)	8 (38%)
Treatment with mycophenolate	0 (0%)	1(5%)
Treatment with azathioprine	2 (22%)	12 (57%)
Patients died during the study period	0	0

The majority of patients were referred to the gastroenterology service due to asymptomatic elevation in liver function tests. Out of the 30 patients, only 2 had liver enzyme levels elevated more than 10 times the upper limit of normal at the time of presentation. About 4 (44%) of the African American patients in our study had no clinical symptoms, while 15 (77%) of the Hispanic patients were asymptomatic at presentation. Abdominal pain was the most common initial presenting symptom. Patients' initial presentations are summarized in Table 3.

**Table 3:** Signs and Symptoms at Initial Presentation

Initial presentation	Number of patients	Percentage
Referred due to abnormal liver function tests	19	63%
Abdominal pain	6	20%
Nausea/vomiting	1	3.3%
Jaundice	2	6.7%
Altered mental status	1	3.3%
Hepatic encephalopathy	1	3.3%
Worsening abdominal distention requiring hospitalization	1	3.3%
Variceal bleeding	2	6.7%

## Discussion

The prevalence of AIH varies worldwide. A comprehensive analysis of 37 studies by Hahn et al. involving over 239 million participants and 55,839 patients with AIH from 18 countries showed that the incidence of AIH was 1.28 per 100,000 inhabitant-years [3]. At the same time, its prevalence was 15.65 cases per 100,000 inhabitants [2,3]. The study also suggests that the prevalence of AIH has been increasing over the years, with noticeable growth from 1970 to 2019. A study done in the United States from April 2014 to April 2019 in 26 healthcare systems found the prevalence of AIH at 31.2/100,000 persons [7]. A lower prevalence was reported by Kim et al. in a study done in South Korea, which found the prevalence of AIH to be at 4.8/100,000 persons [8].

Like other autoimmune diseases, AIH is typically more prevalent in females. The ratio of affected females to males is 4:1 [9,10]. A study done on 1721 patients in Denmark reported that the prevalence of AIH hepatitis in females was 34.6 per 100,000 as compared to 13.0 per 100,000 in males [11]. Tunio et al. [7] a study in the United States with 11,600 individuals reported that the prevalence of autoimmune hepatitis in females was 45.0 per 100,000 as compared to 14.0 per 100,000 in males. In this study, 75% of the patients with AIH were Caucasian, while African American and Hispanic compromised only 14% and 3%, respectively [7]

Previous studies have shown that the age of presentation is bimodal, with a peak in the second decade of life and another peak between the fifth and sixth decade [11, 12]. Baven-Pronk et al. [13] previously observed trends suggestive of less acute presentation in patients with AIH when the age at diagnosis was over 60. They also noted a higher rate of asymptomatic presentation when the disease is onset is between 40 and 70 years old [13]. In our study, the mean age at diagnosis of AIH was 45, suggesting a later onset of signs and symptoms.

The development of AIH is a multifaceted process influenced by both genetics and environmental factors. Genetic predispositions play a significant role, where specific genetic traits can alter immune system responses, leading to an attack on liver cells through molecular mimicry. Critical mediators in the immune system, such as CD4+ T cells and regulatory T cells, become dysfunctional, contributing to the disease progression. Additionally, CD8+ cytotoxic T cells and autoantibody-producing B cells further exacerbate liver damage. Environmental triggers, including drug exposure (such as nitrofurantoin, minocycline, methyldopa, hydralazine), viral infections (hepatitis A, B, C, and E, Epstein-Barr virus), and alterations in the gut microbiome, are also thought to interact with these genetic factors, leading to a complex pathway that culminates in AIH [4, 14, 15].

AIH diagnosis is based on multiple factors, including clinical presentation, biochemical tests, and histology. There are three main types of AIH based on the types of autoantibodies: Type 1, Type 2, and autoantibody-negative autoimmune hepatitis. Type 1 AIH (AIH-1) is characterized by positive ANA or ASMA, making up about 75-80% of AIH cases. It is typically diagnosed in adulthood and generally has slower progression compared to type 2. Type 2 AIH (AIH-2) has the presence of anti-LKM-1; it is usually diagnosed during childhood and constitutes about 10-15% of AIH cases. This is presented in Table 4.

**Table 4:** Autoimmune Hepatitis Subtypes Table Credit:  Faryal Altaf

Autoimmune hepatitis subtypes
Autoimmune hepatitis type 1
Anti-nuclear antibody
Anti-smooth muscle antibody
Anti-actin antibodies
Anti-mitochondrial antibodies
Anti-soluble liver antigen/liver pancreas antibody-antigen
Anti-single-stranded and anti-double-stranded DNA [5]
Atypical perinuclear anti-neutrophil cytoplasmic antibodies [6]
Autoimmune hepatitis type 2
Antibodies to LKM-1
Anti-liver cytosol antibody-1 (ALC-1) [5]
Autoantibody-negative autoimmune hepatitis or cryptogenic

Another marker used in diagnosing AIH is immunoglobulin G (IgG), which reflects the autoimmune activity within the liver. However, approximately 10% of AIH patients do not show elevated IgG levels at diagnosis. The diagnosis is further supported by the presence of other autoimmune conditions like thyroid disease, type 1 diabetes, and vitiligo [6,16-18]. In our study, 23% of patients had a concomitant autoimmune disease.

The International Autoimmune Hepatitis Group (2008) introduced a more straightforward approach to improve diagnostic accuracy. This revised system focuses on four main elements: the histology of the liver, the presence of specific autoantibodies (such as ANA, SMA, anti-LKM1, anti-LKM3, and anti-LC1), the levels of γ-globulin or IgG, and the exclusion of viral hepatitis. These criteria have successfully achieved high specificity and sensitivity for confirming the diagnosis of AIH. Nonetheless, the simplified criteria have not been as effective in excluding AIH in patients with other liver conditions. Therefore, liver biopsy remains fundamental in confirming the disease, as it allows pathologists to observe specific histological features such as interface hepatitis, rosetting of hepatocytes, and lymphoplasmacytic infiltration, which are characteristic of AIH and crucial for its diagnosis [19,20].

AIH can present in diverse ways, from asymptomatic liver enzyme elevations to acute liver failure [2]. Symptoms can vary significantly based on the severity and stage of the disease. About 25% of cases are asymptomatic at the time of presentation. The most common presenting symptoms include fatigue, abdominal pain, and arthralgias. Physical examination may reveal hepatomegaly in approximately 78% of cases and jaundice in approximately 69% of individuals with advanced disease [21]. At the time of diagnosis, about 25% of patients already have cirrhosis [22]. In our study, 63% of the patients presented with abnormal liver function tests and no associated clinical symptoms. Abdominal pain and jaundice were the initial clinical presentation in 20% and 6.7% of patients, respectively. Additionally, 36% of patients required hospitalization due to decompensated liver cirrhosis.

The most common treatment for AIH includes glucocorticoids and azathioprine, and the goal is to achieve normalization of liver tests, prevent further liver damage, and restore immunoglobulin levels. Glucocorticoids interact with glucocorticoid receptors to suppress pro-inflammatory genes and activate anti-inflammatory genes. Azathioprine is a steroid-sparing agent, which is generally started a few weeks after beginning steroids to reduce their side effects by inhibiting purine synthesis and affecting rapidly dividing cells like lymphocytes. For patients who do not respond to or tolerate these first-line treatments, alternative or second-line options like 6-mercaptopurine or mycophenolate mofetil are considered. Additionally, about 10-20% of AIH patients may require third-line therapies such as calcineurin inhibitors or cell-depleting drugs like rituximab. The use of TNF-α inhibitors, including infliximab, has also been explored but comes with risks of complications like drug-induced liver injury [6,20]. In our study, 43% of patients were being treated with oral corticosteroids, 46.67% were using azathioprine, and only 3.3% were receiving mycophenolate, indicating a low rate of secondary or tertiary treatment agents.

The clinical outcomes of AIH can vary significantly across different ethnic groups. Poorer prognoses are seen in Asians, who tend to have worse survival outcomes compared to other ethnic groups [23]. Indigenous Alaskan populations are more likely to present with acute jaundice, while Hispanic patients exhibit the highest prevalence of cirrhosis among AIH patients. Wen et al. [5], in a retrospective analysis using the Nationwide Inpatient Sample (NIS), reported a significant racial disparity in AIH hospitalizations. According to the report, Blacks were hospitalized for AIH at a rate of 69%, which is 20% higher than whites (P < 0.001). Wong et al. [4] performed a retrospective study analyzing patient data from 1999 to 2010 in a large tertiary-care community hospital. Findings showed that 34% of the AIH patients had biopsy-confirmed cirrhosis, with Hispanics showing the highest prevalence at 55%. The analysis also showed that Hispanics had lower serum albumin levels and platelet counts than whites, alongside a higher INR (P values < 0.001 and 0.05, respectively) [4,5,8,14]. In our study, the overall prevalence of cirrhosis was 70%, with 80% of Hispanic patients having cirrhosis.

Lee et al. [24], in a single-center analysis at Zuckerberg San Francisco General Hospital, explored the relationship between race, ethnicity, and AIH. Among 63 AIH patients and 2,049 non-AIH controls, race/ethnicity was a significant predictor of AIH diagnosis. In this study, they concluded that Black (OR 9.6), Latino (OR 25.0), and Asian/Pacific Islander (OR 10.8) individuals had higher odds of being diagnosed with AIH compared to the white reference group [23]. Despite these differences in diagnosis rates, the clinical features of AIH, such as baseline ALT, total bilirubin, fibrosis at presentation, and hospitalization rates, did not significantly differ among these racial groups [24].

Uihwan Lee et al. [25], in a review of the NIS from 2012 to 2017, found that Black individuals had higher rates of acute liver failure (ALF) and hepatorenal syndrome related to ALF but lower rates of cirrhosis-related encephalopathy compared to White individuals. Black individuals also experienced longer hospital stays (aOR 1.071, 95% CI 1.050-1.092). Additionally, it was reported that Hispanic individuals had higher rates of cirrhosis-related complications, including ascites, varices, variceal bleeding, spontaneous bacterial peritonitis, and encephalopathy [26]. Among our study group, 36% of patients needed hospital admission due to decompensated liver cirrhosis.

A retrospective study by de Boer et al. [26] compared the clinical characteristics and outcomes of Black and White patients with AIH. The study reported that Black patients presented at a younger median age (38 vs. 45 years) and had higher mean IgG levels (31.0 mg/dL vs. 27.5 mg/dL) than white patients. While there were no significant differences in autoantibody profiles, International AIH Group scores, or sex distribution, a notable disparity was observed in the prevalence of systemic lupus erythematosus, which was higher in Black patients (10% vs. 2%). Response rates to standard therapy and relapse rates were similar between both groups. However, Black patients exhibited a significantly higher risk of requiring liver transplantation and facing liver-related mortality. Despite these differences, overall mortality rates were comparable between the two ethnic groups [26]. A summary of the results of other studies is presented in Table 5 [1,4,5,24-26].

**Table 5:** Comparison of Various Studies on Clinical Course of Autoimmune Hepatitis Table Credit: Franklin Sosa AIH: autoimmune hepatitis, ICD-9: International Classification of Diseases, Ninth Revision, SLE: systemic lupus erythematosus, IgG: immunoglobulin G, US: United States.

Title	Author name and publication year	Selected population	Patients (n)	Endpoint/tested parameters	Significant results
Clinical characteristics and response to therapy of autoimmune hepatitis in an urban Latino population	Zahiruddin et al., 2016 [1]	Latino patients with AIH diagnosed between 2002 and 2012	28	AIH outcomes in Latino patients	Complete and partial remission were achieved in 71% and 18% of patients, respectively. Additionally, 38% of patients required maintenance prednisone, either alone or in combination with azathioprine or mycophenolate mofetil.
The impact of race/ethnicity on the clinical epidemiology of autoimmune hepatitis	Wong et al., 2012 [4]	Patients diagnosed with AIH from 1999 to 2010 in a large tertiary-care community hospital in New York City	81	AIH epidemiology among a racially diverse population	Hispanic individuals had the highest prevalence of cirrhosis (55%), and Asian individuals had poorer survival outcomes.
Hospitalizations for autoimmune hepatitis disproportionately affect Black and Latino American individuals	Wen et al., 2018 [5]	Patients with primary discharge diagnosis corresponding to the ICD-9 code for AIH in the National Inpatient Sample between 2008 and 2012	9,258	AIH admissions rate of withes vs blacks and Hispanics	Black and Latino individuals were hospitalized for AIH at a rate of 69% (P<0.001) and 20% higher (P<0.001) than White individuals, respectively.
Race/ethnicity is an independent risk factor for autoimmune hepatitis among the San Francisco underserved	Lee et al., 2018 [24]	Patients with AIH, treated at an urban county hospital (The Zuckerberg San Francisco General Hospital) that serves indigent and underresourced communities in San Francisco from January 2002 to June 2017	63 AIH patients and 2,049 control patients	Evaluate the relationship between race/ethnicity and AIH and better characterize its clinical features among different racial groups	Black, Latino, and Asian/Pacific Islander race/ethnicity were associated with increased odds of an AIH diagnosis compared to the White reference group.
Clinical implications of gender and race in patients admitted with autoimmune hepatitis: updated analysis of US hospitals	Lee et al., 2022 [25]	Patients with AIH from the National Inpatient Sample from 2012 to 2017	9,218	Evaluate the effects of race and gender in patients with AIH using national hospital data, specifically focusing on hepatic and extrahepatic comorbidities that result in hospital admission	Black and Hispanic individuals had higher rates of hepatic complications, including ascites, variceal bleeding, spontaneous bacterial peritonitis, and encephalopathy.
Association between Black race and presentation and liver-related outcomes of patients with autoimmune hepatitis	de Boer et al., 2019 [26]	Patients with AIH attending the Institute of Liver Studies at King's College Hospital, London (1971-October 2015), the Royal Free Hospital, London (1982–2016), and the multicenter Dutch Autoimmune Hepatitis Study Group cohort (2006-August 2016)	88 Black patients and 897 White patients with AIH	To characterize the presentation and outcome in Black and White patients with AIH	Black patients present at a younger age, have higher levels of IgG levels, and have a higher proportion of SLE. Also, Black individuals have a higher risk of liver transplantation and liver-related mortality.

These studies show that further research is needed to identify risk factors that may be related to the higher risk of morbidity and mortality in Black and Hispanic patients. Addressing these risk factors may create opportunities for implementing interventions to reduce healthcare disparities.

Limitations of our study include a small sample size, despite the long duration of the retrospective study. This is likely due to the low incidence of autoimmune hepatitis in the African American and Hispanic populations. Due to the small sample size, statistical analysis of our study results could not be performed.

## Conclusions

Hispanic patients were more likely to be diagnosed at an older age compared to African American patients. The majority of our patients developed liver cirrhosis, and a significant number required hospitalization due to decompensated liver disease. Hispanic patients were more likely to develop liver cirrhosis compared to African American patients. They were also more likely to be asymptomatic at the time of diagnosis. Our study highlights the importance of conducting targeted research to enhance the understanding of AIH in African American and Hispanic populations.
